# Short-Term Outcomes of Cementless Total Hip Arthroplasty Using a 3D-Printed Acetabular Cup Manufactured by Directed Energy Deposition: A Prospective Observational Study

**DOI:** 10.3390/jcm14134527

**Published:** 2025-06-26

**Authors:** Ji Hoon Bahk, Woo-Lam Jo, Kee-Haeng Lee, Joo-Hyoun Song, Seung-Chan Kim, Young Wook Lim

**Affiliations:** 1 Department of Orthopedic Surgery, Bucheon St. Mary’s Hospital, College of Medicine, The Catholic University of Korea, Seoul 06591, Republic of Korea; tonybahk@gmail.com (J.H.B.);; 2 Department of Orthopedic Surgery, Seoul St. Mary’s Hospital, College of Medicine, The Catholic University of Korea, Seoul 06591, Republic of Korea; 3 Department of Orthopedic Surgery, St. Vincent’s Hospital, College of Medicine, The Catholic University of Korea, Seoul 06591, Republic of Korea; 4 Department of Orthopedic Surgery, Eunpyeong St. Mary’s Hospital, College of Medicine, The Catholic University of Korea, Seoul 06591, Republic of Korea

**Keywords:** total hip arthroplasty, cementless acetabular cup, directed energy deposition, additive manufacturing, osseointegration, 3D printing

## Abstract

**Background/Objectives**: Additive manufacturing (AM) enables the production of cementless acetabular cups with porous surfaces that facilitate early osseointegration. Directed energy deposition (DED), a form of AM, allows the direct welding of porous structures onto metal substrates without requiring a vacuum environment, offering advantages over conventional powder bed fusion methods. Despite growing interest in DED, no prospective clinical studies evaluating DED-based acetabular components have been published to date. This study assessed short-term outcomes of a DED-based 3D-printed acetabular cup in total hip arthroplasty (THA). **Methods**: A total of 120 patients who underwent primary cementless THA using the Corentec Mirabo Z^®^ acetabular cup were prospectively enrolled. Among them, 124 hips from 100 patients who had completed a minimum of 24 months of follow-up were included in the analysis. Clinical outcomes were assessed using the Harris hip score (HHS), WOMAC, EQ-5D-5L, and pain NRS. Radiographic evaluation included measurements of cup position, osseointegration, and detection of interfacial or polar gaps on CT and plain radiographs. Implant-related complications were also recorded. **Results**: At a mean follow-up of 34.6 months, the implant survival rate was 99.3%, with one revision due to suspected osseointegration failure. The HHS improved from 56.6 to 91.4 at 24 months, and the NRS decreased from 6.2 to 1.1 (both *p* < 0.001). Interfacial gaps were observed in 58.1% of cases on CT, though most were <1 mm and not clinically significant. Common postoperative issues included greater trochanteric pain syndrome, squeaking, and iliotibial band tightness, all of which were resolved with conservative treatment. **Conclusions**: DED-based 3D-printed acetabular cups demonstrated favorable short-term clinical and radiographic outcomes, with high survivorship and reliable early osseointegration in cementless THA.

## 1. Introduction

The surface coatings of cementless acetabular cups in total hip arthroplasty (THA) have been developed primarily to promote rapid osseointegration, which is critical for achieving long-term secondary stability following successful initial press-fit fixation. Titanium plasma spray (TPS) coating has been widely used for many years. However, technical limitations such as potential delamination and concerns regarding long-term mechanical integrity have been raised. In recent years, there has been a growing shift toward 3D-printed acetabular cups. Additive manufacturing (AM), including widely adopted powder bed fusion (PBF) methods such as electron beam melting (EBM) [1] and selective laser melting (SLM) [2], enables the production of acetabular components with porous surfaces that mimic trabecular bone, promoting osseointegration in cementless THA [3]. Short-term studies have shown promising clinical outcomes with AM-based implants [4,5,6]. However, limitations of PBF include environmental sensitivity, powder bed size constraints, and the potential for anisotropic mechanical properties and internal defects that may affect implant reliability [7].

On the other hand, directed energy deposition (DED) is a 3D metal printing technique that integrates porous structures directly onto a metal surface to effectively replicate the trabecular architecture of cancellous bone, thereby promoting bone ingrowth [8,9]. Direct metal fabrication (DMF), also known as direct metal tooling (DMT), is a DED-based method in which metal powders, such as titanium, are fed into a laser beam that melts the target surface and simultaneously welds the material to the substrate. This process enables the direct deposition of porous structures even on curved geometries. The technique also allows for the optimization of surface porosity, typically around 30%, with pore sizes ranging from 50 to 200 μm. These parameters are considered favorable for enhancing both initial press-fit stability and long-term biological fixation. The biological and mechanical properties of DMT-based surface coatings have been evaluated in both in vitro and in vivo settings [9,10,11], although prospective observational studies are currently lacking.

Compared to other AM techniques, such as PBF, DED offers several advantages. It does not require a vacuum environment, enables lower production costs, and, most importantly, permits direct welding of dissimilar metals onto the base cup without forming a separate coating layer, thereby reducing the risk of delamination. In addition, this approach improves manufacturing efficiency by allowing mass production of standard titanium cup bases. These can subsequently be modified with porous surfaces using DED, even when different types of biocompatible metals are required [8,9,10,11,12]. A concise comparison of DED and PBF techniques is summarized in Table 1.

Corentec Mirabo Z^®^ is a novel, commercially available acetabular component manufactured using DMT technology. In this study, we prospectively evaluated patients who underwent cementless THA using this implant. To our knowledge, this is the first prospective short-term observational study to investigate acetabular components produced using DED technology. The purpose of this study was to assess the radiological and clinical outcomes with a minimum follow-up of two years.

## 2. Materials and Methods

### 2.1. Patient Cohort Characteristics and Follow-Up Status

This single-center prospective study was approved by the Institutional Review Board of this institute (Protocol code: XC21OIDI0006; 27 April 2021) prior to initiation and was conducted and reported in accordance with the STROBE (strengthening the reporting of observational studies in epidemiology) guidelines. Consecutive patients who underwent primary THAs for hip disease from March 2021 to January 2023 were assessed for eligibility, and in total, 144 hips from 120 patients were enrolled in the study. DED-based 3D-printed acetabular cups were implanted in the subject patients and were followed for a minimum of 24 months. Traumatic causes, including fractures, were excluded from enrollment. Plain hip radiographs and clinical outcomes were evaluated over a minimum two-year follow-up, which concluded in January 2024 for the last enrolled patient.

**Figure 1:**
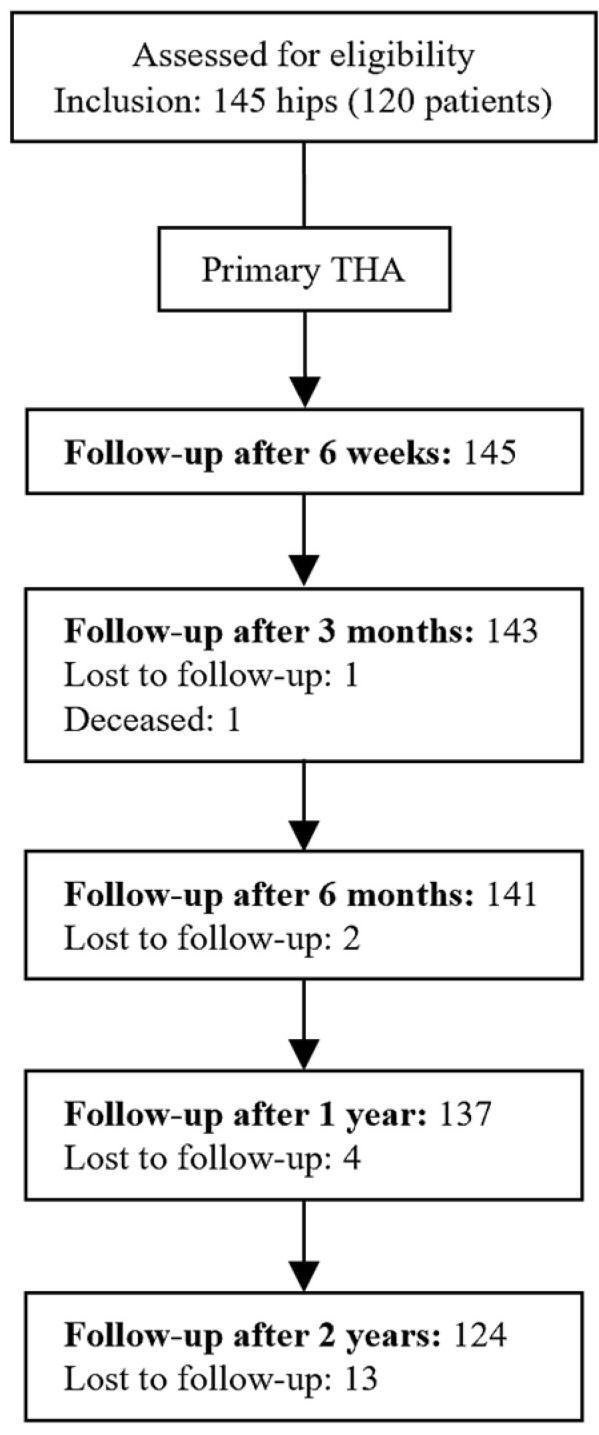
Flow diagram of patient follow-up and completion after index primary total hip arthroplasty.

At a 3-month follow-up, one patient died due to exacerbation of bronchiolitis obliterans organizing pneumonia (BOOP) as a consequence of worsening underlying acute myeloid leukemia. A total of 20 patients (21 hips) were lost to follow-up during the 24-month follow-up period, with a total of 124 hips remaining eligible for final analysis (Figure 1). After 24 months, patients were kept on follow-up, and as of the time of analysis, 47 hips had been followed up to 36 months. At 26 months, a patient died of BOOP exacerbation associated with preexisting acute lymphoid leukemia. Characteristics of patients who completed 24-month follow-up are summarized in Table 2. Lost to follow-up patients were excluded from the final analysis.

### 2.2. Implant Properties

**Figure 2:**
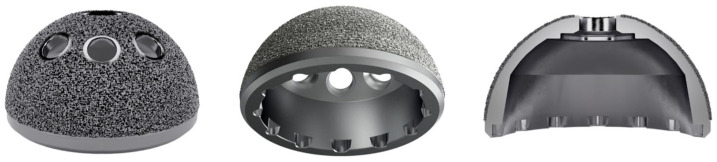
Photograph and schematic diagram of the acetabular cup used in this prospective cohort.

The Corentec Bencox Mirabo Z^®^ (Corentec Co., Ltd., Cheonan, Republic of Korea) is a recently introduced 3D-printed cementless acetabular cup that is commercially available, manufactured using DMT AM technology designated as Cortinium or Z coating (Figure 2). The cup offers three acetabular screw holes with a slimmed peripheral rim. Femoral components were selected based on each patient’s proximal femoral geometry and clinical characteristics.

### 2.3. Surgical Procedure and Perioperative Patient Care

All THAs were conducted by a senior high-volume arthroplasty surgeon in a single center. A posterolateral approach was used in all cases, with the subject acetabular cups and cementless femoral components assembled with fourth-generation ceramic-on-ceramic articulations. All press-fit acetabular cups were inserted after 1 mm underreaming. In cases with advanced osteoarthritis with large subchondral cysts, the cysts were removed by curettage. Autogenous bone harvested during final reaming was grafted and reverse-reamed for impaction just before cup insertion. Implants used for patients in this study are specified in Table 3.

To reduce surgical stress and promote faster postoperative recovery, the principles of enhanced recovery after surgery (ERAS) were applied [13]. Spinal anesthesia using heavy bupivacaine was preferred over general endotracheal anesthesia, and patients were educated on smoking cessation and limiting alcohol intake. A clear, carbohydrate-rich beverage was allowed up to two hours before surgery. The protocol also included the routine use of intermittent pneumatic compression, early removal of urinary catheters, and early mobilization with walkers. Intravenous prophylactic antibiotics (cefazolin) were administered prior to skin incision and maintained for 24 h postoperatively. Pharmacologic antithrombotic agents were not routinely used. A local cocktail injection containing cefazolin (1 g), ketorolac (30 mg), ropivacaine (40 mg), and epinephrine (1 mg) diluted in normal saline was locally injected into the pericapsular structures, preserved short external rotators, gluteus maximus, and subcutaneous tissues before wound closure. Additionally, tranexamic acid (1 g) was locally injected into the quadratus femoris muscle to reduce postoperative bleeding. Postoperative analgesia was managed with fentanyl-based patient-controlled analgesia (PCA) and selective COX-2 inhibitors, both administered unless contraindicated. A standardized clinical pathway was applied to all patients, with routine discharge on postoperative day 5 (hospital day 7) in the absence of medical or surgical complications.

All procedures were performed using a posterolateral approach with the patient in the lateral decubitus position. The short external rotator preservation technique was used, involving capsulectomy and preservation of the obturator internus. Therefore, no additional tendon repair was necessary during wound closure [14]. Walker-assisted ambulation with full weight-bearing was initiated on the first postoperative day, and discharge was planned for postoperative day five, with an average hospital stay of 7.16 days per hip.

### 2.4. Radiological Evaluation

Prior to surgery, standard hip anteroposterior (AP) templating, lateral, and trans-lateral radiographs were obtained for baseline assessment and preoperative planning. Templating radiographs were acquired with the pelvis centered at the pubic symphysis, and both hips were manually internally rotated by 10° to 15° to achieve a true AP view of the proximal femurs. All preoperative templating radiographs and the postoperative true AP hip radiograph obtained three days after surgery were taken under the direct supervision of a single orthopedic surgeon, who confirmed the adequacy of each image on site.

A nonenhanced three-dimensional pelvic bone CT (SOMATOM Force, Siemens Healthineers, Erlangen, Germany; 120 kVp, 1.0 mm slice thickness) was obtained on postoperative day 3 for precise evaluation of the cup configuration and acetabular gaps. The metal artifact reduction algorithms (Iterative Metal Artifact Reduction (iMAR), Siemens Healthcare, Germany) were applied to postoperative CTs upon request to the radiology department prior to the scans. iMAR is a postprocessing technique that combines beam hardening correction, normalized sinogram inpainting, and frequency split within an iterative reconstruction loop [15,16]. Polar gaps were measured as the distance between the pole of the implant and the corresponding reamed acetabular cavity on axial CT images and hip AP radiographs using the digital caliper tool (nU PACS version 1.0.0.42.1, Anyang, Republic of Korea). In addition, even minimal interface gaps in three zones (A, B, and C) defined by DeLee and Charnley [17] were evaluated, with zone classification not mutually exclusive such that gaps could be assigned to multiple zones. All images were reviewed by two orthopedic hip arthroplasty surgeons in identical conditions (window width: 380 HU, window center: 2200 HU) for coronal and axial views. The inclination of an implanted acetabular cup was measured on a properly positioned hip AP radiograph, while the anteversion was measured using axial CT images. Anteversion was defined as the angle between the opening plane of the cup and a reference line perpendicular to the line connecting the anterior and posterior acetabular ridges at the level of the sciatic notch [18,19]. To evaluate interobserver agreement, 50 out of 124 cases were randomly selected for independent measurement. One of the surgeons re-evaluated the previously assessed cases after a 3-week interval to assess intra-observer agreement, blinded to the initial assessment.

Postoperative follow-up visits were scheduled at 6 weeks, 3 months, 6 months, 1 year, and annually thereafter. Radiographs were obtained at each follow-up visit, and the images were assessed for the extent of osseointegration between the acetabular cup and the reamed acetabular surface, as well as for the presence of subclinical periprosthetic fractures that may have occurred during cup implantation. Follow-up radiographs were also evaluated for signs of osteolysis, component loosening, periprosthetic fractures, and heterotopic ossification. Single-photon emission computed tomography (SPECT) was additionally used in cases where ongoing osteolysis was suspected.

### 2.5. Patient-Reported Outcome Measures and Clinical Evaluation

Patient-reported outcome measures (PROMs) were evaluated at each follow-up visit using self-administered questionnaires. These included the Harris hip score (HHS), the EuroQol-5D-5L (EQ-5D), the Western Ontario and McMaster Universities Osteoarthritis Index (WOMAC), and the numeric rating scale (NRS) for pain. The HHS (range: 0–100) assesses hip function, with higher scores indicating better function. The EQ-5D-5L evaluates five dimensions: mobility, self-care, usual activities, pain/discomfort, and anxiety/depression. The EQ-5D index score, calculated using the national value set, yields a utility value between 0 (representing death) and 1 (representing perfect health), where higher scores reflect better health-related quality of life. The WOMAC score (range: 0–96) measures pain, stiffness, and physical function; higher scores indicate greater symptom severity. Pain intensity was assessed using the NRS (range: 0–10), where higher scores correspond to more severe pain.

At each follow-up, patients were also assessed for the presence of audible noises, such as squeaking, clicking, clunking, or grinding, as well as any pain or discomfort. When pain was reported, a focused physical examination followed, including Patrick’s test, palpation for gluteal tendon tenderness, assessment of tenderness in the upper outer quadrant of the buttock, and evaluation of iliotibial band tightness. For patients diagnosed with greater trochanteric pain syndrome (GTPS), iliotibial band tightness, or iliopsoas impingement, ultrasound-guided injections were administered using 40 mg of triamcinolone combined with 1 mL of 2% lidocaine.

### 2.6. Sample Size Calculation and Statistical Analysis

The sample size was calculated using G*Power 3.1.9.4 (Heinrich Heine University, Düsseldorf, Germany) for a paired t-test with a two-tailed α of 0.05, power of 0.95, and a medium effect size (Cohen’s d = 0.5), indicating that a minimum of 54 cases was required. To account for an anticipated 20% attrition, including loss to follow-up and patient death, 120 patients were enrolled to ensure sufficient statistical power, given that the study was initiated during the COVID-19 pandemic. Preoperative and follow-up scores were compared using paired t-tests conducted with the Statistical Package for the Social Sciences (SPSS), version 26 (SPSS Inc., Chicago, IL, USA). GraphPad Prism, version 10.4.1 (GraphPad Software Inc., San Diego, CA, USA) was used to generate the PROM score plots and perform the Kaplan–Meier survival analysis. The normality of the differences between preoperative and postoperative values was assessed using the Shapiro–Wilk test and confirmed across all time-point comparisons. Bonferroni correction was applied to adjust for multiple comparisons involving preoperative values at two postoperative time points (24 and 36 months), with an adjusted significance threshold of *p* < 0.025.

## 3. Results

### 3.1. Follow-Up Rate and Implant Survival

**Figure 3:**
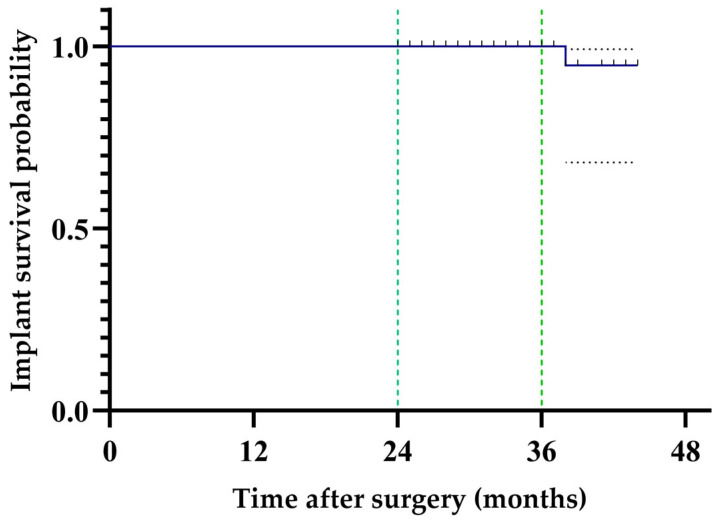
Kaplan–Meier survival curve following cementless THA using DMT-based 3D-printed acetabular cups.

Among 124 hips from 100 patients who completed the minimum follow-up period, the follow-up rate was 85.5%. The implant survival rate during the follow-up period (mean 34.6 months) was 99.3%, with one case requiring revision at 38 months due to failure to achieve sufficient osseointegration, which might have resulted from repetitive abnormal mechanical loading caused by progressive degenerative sagittal imbalance leading to spinopelvic instability (Figure 3).

### 3.2. Radiological Evaluation

Upon postoperative radiograph measurement, the mean acetabular cup inclination was 44.1 ± 6.00° (24.0–58.0°) and the mean anteversion was 32.0 ± 9.06° (0.9–62.0°). Measurements from CT scans taken on postoperative day 3 revealed polar gaps in 4.0% of cases (5/124), ranging from 1.5 to 3.0 mm. Interfacial gaps, strictly defined as any visible separation, were found in 58.1% (72/124), of which 69.4% (50/72) had gaps smaller than 1 mm. Among the 22 cases with gaps greater than 1 mm, 45.5% (10/22) involved zone B and 86.4% (19/22) involved zone C. When all gaps, including those under 1 mm, were considered, 2.8% (2/72) were present in zone A, 81.2% (59/72) in zone B, and 94.4% (68/72) in zone C. Additionally, these findings were concordantly observed in plain hip AP radiographs in only 79.1% (57/72) of the cases. Cohen’s kappa values for intra- and interobserver agreement were 0.89 and 0.80, respectively.

### 3.3. Clinical Evaluation: Postoperative Complications

Complications directly related to acetabular cups observed during the follow-up period included a case of iliopsoas impingement treated with triamcinolone and lidocaine injection, a case of posterior dislocation treated with manual reduction and a hip abduction brace, and a case of aseptic loosening that required revision. Among other complications, greater trochanteric pain syndrome (GTPS) was most commonly observed (5.5%), followed by squeaking (4.8%), iliotibial band (ITB) tightness (3.4%), and anterior thigh pain (2.0%) (Table 4). When GTPS or ITB tightness was identified at follow-up, the patient was immediately treated with an ultrasonography-guided triamcinolone and lidocaine injection in the outpatient clinic, with all 15 cases demonstrating complete resolution of symptoms at the final follow-up.

### 3.4. Clinical Evaluation: PROMs

**Figure 4:**
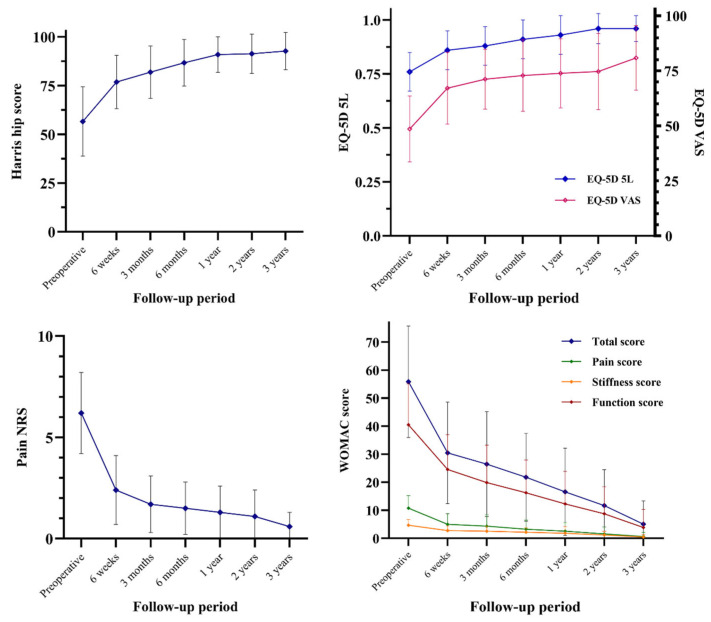
Changes in patient-reported outcome measures over time, including Harris hip score, pain NRS, EQ-5D-5L, and WOMAC, from preoperative baseline to 3 years postoperatively.

Clinical outcomes improved significantly over time. The HHS increased from 56.6 ± 17.8 preoperatively to 91.4 ± 10.1 at 24 months (*p* < 0.001) and further to 92.7 ± 9.6 at 36 months. The pain NRS decreased from 6.2 ± 2.0 to 1.1 ± 1.3 at 24 months (*p* < 0.001) and to 0.6 ± 0.7 at 36 months. The EQ-5D-5L score improved from 0.76 ± 0.09 at baseline to 0.96 ± 0.07 at 24 months (*p* < 0.001) and remained stable at 0.96 ± 0.06 at 36 months. Similarly, the WOMAC score showed marked improvement, decreasing from 55.9 ± 19.9 preoperatively to 11.7 ± 12.8 at 24 months (*p* < 0.001) and to 5.1 ± 8.3 at 36 months (Figure 4).

## 4. Discussion

Among DED-coated acetabular shells, distinctive design features of the Mirabo Z^®^ cup include a slimmed cup rim to minimize iliopsoas tendon impingement, a slightly flattened dome to enhance physiological loading, and enhanced robustness of inner diameter roundness after ceramic liner insertion compared to TPS-treated counterparts, minimizing the risk of ceramic liner fractures [12]. The Mirabo Z^®^ acetabular shell was available in outer diameters of 50 mm to 56 mm during this prospective study, along with compatible fourth-generation ceramic liners (BIOLOX^®^ Delta; CeramTec AG, Plochingen, Germany) or ultra-high molecular weight polyethylene liner options. This study did not limit the type of liners, but ceramic liners were used in all cases in this cohort, reflecting the regional preference for ceramic-on-ceramic THAs. Additionally, the product did not offer dual mobility options at the time of the index surgeries, which limited implant selection in patients with degenerative sagittal imbalance or fused spine.

The clinical and radiological outcomes in this study cohort were comparable to those reported in other short-term observational studies of primary THA using cementless 3D-printed cups manufactured by EBM [20,21,22,23,24] or SLM [25,26,27], which have shown implant survival rates ranging from 99.1% to 100%. The implant survival rate in the present study was 99.3%, consistent with these findings. The incidence of polar gaps or interfacial gaps in zone B following implantation of cementless acetabular components has been reported to range from 1.0% to 36.07% in previous studies [28,29,30]. However, the measurement criteria varied across studies, with gap thresholds ranging from 0.5 mm to 1.0 mm, depending on imaging detectability and definitions set by individual investigators. In a biomechanical study, MacKenzie et al. reported a polar gap incidence of 8.8% and an overall gap incidence of 12.3% in line-to-line press-fit cups [31]. In the present study, the incidence of polar gaps was 4.0%, and interfacial gaps were observed in 58.1% of cases, which appears higher than previously reported values. This discrepancy may be attributed to differences in imaging modality, as our study utilized high-resolution CT for gap assessment, which may have been more sensitive compared to conventional radiographs. Furthermore, even minimal gaps were evaluated, and overlapping zone classification may have contributed to the higher detection rate. Despite the relatively high frequency of interfacial gaps, the majority (69.4%) measured less than 1 mm, which may not be clinically significant [5].

Despite the relatively high frequency of interfacial gaps, most were minimal and not clinically significant. Importantly, patient-reported outcome measures showed consistent and significant improvement throughout the follow-up period. This upward trend was maintained even though some late-onset symptoms such as GTPS, ITB tightness, or aggravation of spinal stenosis were included in the dataset. However, a limitation of PROMs is that pain NRS, EQ-5D, WOMAC, and even Harris hip scores can be influenced by comorbid medical conditions. Spinal stenosis and knee arthritis are the most common confounders affecting pain and function scores, even when the focus is limited to hip status. Nevertheless, the statistical significance of trends observed in these PROMs remains meaningful in an observational study of an implant. Considering these limitations, the mean HHS values in this cohort still appear comparable to those reported in a recent meta-analysis of randomized controlled trials using posterior approaches regardless of implant type, which showed average HHS scores of 82.75 at 3 months, 88.45 at 6 months, and 92.37 at 12 months [32].

**Figure 5:**
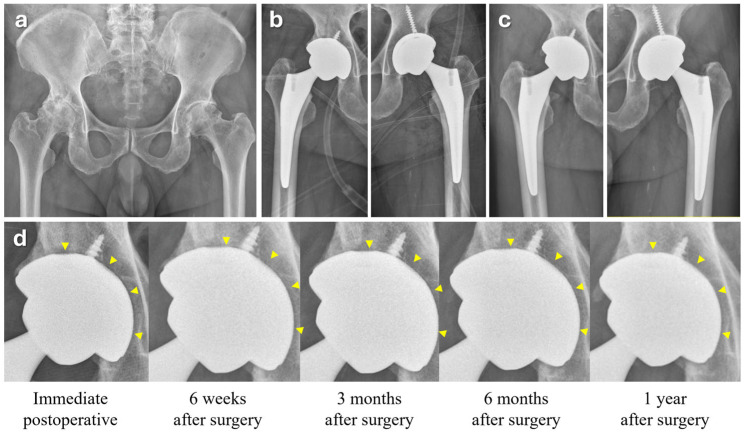
Serial radiographs following total hip arthroplasty using a 3D-printed acetabular component. (**a**) Preoperative hip AP radiograph showing bilateral advanced osteoarthritis in the hip joints. (**b**,**c**) Serial AP radiographs of the operated hips from immediately after surgery to 4 years postoperatively. (**d**) Magnified views of the acetabular component at each time point. Yellow arrowheads indicate the radiolucent line between the cup and the host bone. The gap observed immediately after surgery gradually decreased over time, becoming undetectable from 6 months postoperatively, suggesting progressive osseointegration of the implant.

A successful osseous integration around the acetabular cup was confirmed in a representative case (Figure 5). A 78-year-old female with bilateral advanced hip osteoarthritis was treated with staggered THA at a one-week interval between procedures. A slight gap around the entire cup–acetabular interface was clearly visible in the immediate postoperative hip AP radiograph, which was most pronounced at the pole of the cup, forming a polar gap. Throughout follow-up, this gap gradually decreased due to acetabular bone remodeling and ingrowth, which was evident in the radiograph taken six months after surgery. PROM scores peaked one year postoperatively, and the patient remained pain-free and satisfied at the final follow-up, 44 months after surgery.

**Figure 6:**
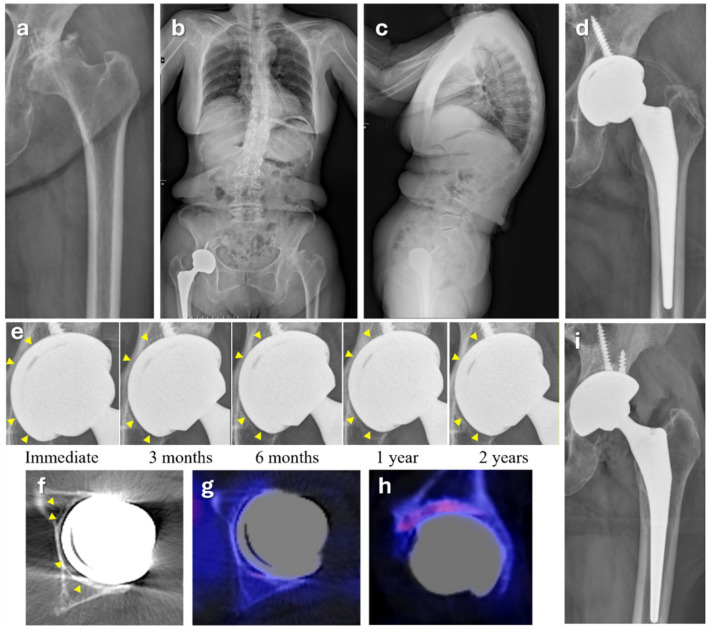
Radiographic and nuclear imaging evaluation of a patient with suspected failure of osseointegration after THA. (**a**) Preoperative anteroposterior radiograph showing severe arthritic changes in the hip joint with femoral head destruction. (**b**,**c**) Standing anteroposterior and lateral whole-spine radiographs demonstrating degenerative spinal imbalance. (**d**) Immediate postoperative radiograph of the left hip. (**e**) Serial magnified radiographs of the acetabular component from immediately after surgery to 2 years postoperatively. Yellow arrowheads indicate persistent radiolucent lines around the cup–bone interface, suggesting a lack of osseointegration. (**f**–**h**) SPECT/CT images revealing a radiolucent gap around the acetabular component, with increased uptake at the superior aspect of the acetabular component, indicating mechanical loosening. (**i**) Postoperative radiograph after revision.

On the other hand, one case required revision due to aseptic loosening associated with changes in spinopelvic dissociation after surgery (Figure 6). A 74-year-old female with advanced secondary osteoarthritis due to hip dysplasia was treated with THA. Preoperative standing whole-spine radiographs showed a markedly elevated pelvic incidence, possibly overestimated due to a flexed knee, hyperextended spine, and retroverted pelvis as compensation for degenerative sagittal imbalance. Fixed-bearing THA was performed in a hip with severe arthritic changes, and initial mechanical stability was achieved. However, failure to achieve secondary stability through osseointegration or fibrous fixation was suspected during follow-up up to three years postoperatively, which may have been caused by repetitive spinopelvic instability. SPECT revealed uptakes in the acetabular sourcil and a diffuse fibrous gap between the bone and the cup surface. It was then revised with a multi-hole dual mobility cup of the same size from the same product line, which was later released commercially.

This study has several limitations. First, it was a prospective observational study rather than a comparative trial and, therefore, cannot demonstrate the superiority of the DED-based acetabular component over other AM-based 3D-printed shells. Future prospective randomized clinical trials are needed to address this limitation. Radiographic evaluation complemented by biomechanical data, along with serial CT imaging at each follow-up to confirm the timing of bone ingrowth, would further strengthen the validity of the findings. Second, the follow-up rate was lower than typically expected for a study with a minimum two-year follow-up period. This was partly due to the COVID-19 pandemic, which disrupted routine outpatient visits. Additionally, as the study was conducted at a major tertiary referral center, many patients had traveled long distances for surgery and were less likely to return for follow-up, particularly in the absence of symptoms. Third, bone mineral density (BMD), a factor that may influence acetabular cup stability, was not routinely assessed. Given the relatively young mean age of the cohort (55.1 years), BMD evaluation was not included in the study protocol. Furthermore, as the study was limited to ceramic-on-ceramic bearings, the findings may not be generalizable to other bearing types. Future studies comparing DED and PBF cups across various bearing surfaces with longer-term follow-up are needed.

## 5. Conclusions

This prospective study demonstrated favorable short-term clinical and radiological outcomes following THA using cementless acetabular cups manufactured with DED-based 3D printing. The implant showed favorable survivorship at a mean follow-up of 34.6 months, with consistent improvements in all patient-reported outcome measures. Although interfacial gaps were frequently detected on high-resolution CT scans, which did not clearly show in the plain radiograph, most were minimal and not clinically significant. These findings suggest that DMT-applied porous coatings may provide reliable fixation and early osseointegration, warranting further investigation in long-term and comparative studies.

## Figures and Tables

**Table 1:** Comparison of DED and PBF methods in acetabular cup manufacturing.

Aspect	PBF	DED
Surface integration	Layer-by-layer porous structures on the cup surface	Directly welded onto the base cup surface (no separate coating)
Environment	Vacuum or controlled	Open-air
Cost-efficiency	Lower	Higher
Geometric compatibility	Limited by powder bed size	Effective for curved surfaces
Mechanical integrity	Anisotropy and potential internal defects	Isotropic, fewer internal defects
Clinical data	Favorable short-term results	Limited

**Table 2:** Demographic characteristics of patients included in the analysis.

Parameter	Total (%)
**Patients (n = 100)**	
Sex	
Male	48 (48)
Female	52 (52)
Follow-up (months)	34.6 (24–44, 8.7)
Age at THA (years)	55.1 (24–77, 12.5)
Body mass index (kg/m^2^)	24.0 (17.1–31.2, 3.0)
**Hips (n = 124)**	
Preoperative diagnosis for primary THA	
Osteonecrosis of the femoral head	77 (62.1)
Primary osteoarthritis	22 (17.7)
Secondary osteoarthritis	21 (16.9)
Dysplasia	11 (8.9)
Post-traumatic	3 (2.4)
Legg-Calvé-Perthes disease sequelae	3 (2.4)
Ankylosing spondylitis	3 (2.4)
Idiopathic coxa vara	1 (0.8)
Subchondral insufficiency fracture	2 (1.6)
Synovial chondromatosis	1 (0.8)
Rheumatoid arthritis	1 (0.8)
Type of anesthesia	
Regional (spinal) anesthesia	73 (58.9)
General endotracheal anesthesia	51 (41.1)
Length of hospital stay	
Unilateral THA	7.16 (7–14, 1.00)
Staggered bilateral THA	14.18 (11–22, 2.43)

**Table 3:** Implant characteristics in the study population undergoing index primary total hip arthroplasty.

Parameter	Total (n = 124)
Cup size	
50 mm	31 (25.0%)
52 mm	45 (36.3%)
54 mm	25 (20.2%)
56 mm	23 (18.5%)
Type of liner	
Ceramic (fourth-generation)	124 (100%)
Ceramic head size	
32 mm	31 (25.0%)
36 mm	93 (75.0%)
Neck length	
Extra-short	3 (2.4%)
Short	41 (33.1%)
Medium	56 (45.2%)
Long	22 (17.7%)
Extra-long	2 (1.6%)
Number of acetabular screws	
0	1 (0.8%)
1	31 (25.0%)
2	92 (74.2%)
Type of femoral component	
Corentec ID^®^	78 (62.9%)
Corentec Bencox II^®^	2 (1.6%)
Stryker Accolade^®^ 127°	37 (29.8%)
Stryker Accolade^®^ 132°	5 (4.0%)
Biomet Taperloc^®^	2 (1.6%)

**Table 4:** Implant-related complications and their mean time to onset following index total hip arthroplasty.

Implant-Related Complications	Hips (%)	Mean Time to Onset (Months, Range)
Greater trochanteric pain syndrome	8 (5.5%)	10 (2–24)
Squeaking	7 (4.8%)	21.8 (11–24)
Iliotibial band tightness	5 (3.4%)	9.3 (3–21)
Anterior thigh pain	3 (2.0%)	7.3 (4–9)
Iliopsoas impingement ^1^	1 (0.7%)	3
Dislocation ^1^	1 (0.7%)	1
Failure of osseointegration ^1^	1 (0.7%)	-
Superficial infection	1 (0.7%)	0.5
Wound dehiscence	1 (0.7%)	0.5

^1^ Acetbaular component-related complications.

## Data Availability

The datasets used and analyses are available from the corresponding author upon reasonable request.
